# Migration of double-J ureteral stent in patients with ureteroileal anastomosis stricture undergoing radical cystectomy and orthotopic neobladder: Analysis risk factors of stent migration

**DOI:** 10.1097/MD.0000000000037765

**Published:** 2024-04-19

**Authors:** Chang Hoon Oh, Soo Buem Cho, Hyo Jeong Lee, Hyeyoung Kwon, Yeok Gu Hwang

**Affiliations:** a Department of Radiology, Ewha Womans University Mokdong Hospital, College of Medicine, Ewha Womans University, Seoul, Republic of Korea; b Department of Radiology, Ewha Womans University Seoul Hospital, College of Medicine, Ewha Womans University, Seoul, Republic of Korea; c Department of Radiology, Chungnam National University Hospital, Chungnam National Uvinersity School of Medicine, Daejeon, Republic of Korea; d Department of Orthopedic Surgery, Ewha Womans University Seoul Hospital, College of Medicine, Ewha Womans University, Seoul, Republic of Korea.

**Keywords:** “Double-J ureteral Stent, Migration”, orthotopic neobladder, radical cystectomy

## Abstract

The objective was to evaluate the incidence and degree of double-J ureteral stent (DJUS) migration. Additionally, we aimed to investigate the risk factors associated with stent migration in the orthotopic neobladder group. In this retrospective study, 61 consecutive patients were included; 35 patients (45 DJUS placements) underwent radical cystectomy with orthotopic neobladder and 26 patients (35 DJUS placements) underwent urinary bladder without cystectomy between July 2021 and March 2023. All the patients were treated with a DJUS for ureteric strictures. The technical success rate was 100% in each group. The DJUS migration was significantly higher in the orthotopic neobladder group, with 22 of 45 cases (48.9%), compared to the urinary bladder group, which had 4 of 35 cases (11.4%) (*P* ≤ .001). Among the patients in the orthotopic neobladder group who experienced DJUS migration, stent dysfunction occurred in 18 cases (81.8%), which was statistically significant (*P *= .003). Multivariate logistic regression analysis revealed that only the size of the DJUS was significantly and positively associated with migration (odds ratio:10.214, *P* = .010). DJUS migration can easily occur in patients undergoing radical cystectomy and orthotopic neobladder, and smaller stent sizes are associated with a higher incidence of migration.

## 1. Introduction

Formation of orthotopic neobladder is frequently performed for urinary diversion following radical cystectomy in patients with bladder cancer. Moreover, an orthotopic neobladder often results in an improved quality of life when compared with an ileal conduit.^[1]^ However, orthotopic neobladders can result in postoperative complications (25%–35%), among which benign ureteroileal anastomotic stricture (UIAS) constitutes a significant proportion (1%–30%).^[2–4]^

Benign UIAS is typically observed 6 to 18 months following surgery. Certain factors, including intraoperative or postoperative blood transfusion, postoperative urinary tract infection, and extracorporeal neobladder anastomoses, can heighten the risk of developing benign UIAS.^[5,6]^ Placement of an internal double-J ureteral stent (DJUS) after radical cystectomy and orthotopic neobladder surgery can reduce the incidence of benign UIAS. However, if UIAS occurs after removal of the DJUS, reinserting an internal ureteral stent is crucial for relieving the urinary system and preventing functional obstruction or even renal failure.^[7]^

Despite the DJUS being designed with pigtail-shaped loops in opposite directions at both the renal and vesical ends to prevent migration, displacement of ureteric stents may still occur. This migration can be either distal or proximal, with the latter being less common with a reported incidence of 1% to 4.2%.^[8]^ Migration of DJUS can occur in various situations, including short stent length, long duration of stent placement without changes, renal movement by respiration, and malpositioning during stent insertion.^[9,10]^

Despite the increasing annual prevalence of bladder cancer, research on the migration and patency of DJUS in patients who have undergone radical cystectomy and orthotopic neobladders is lacking. Therefore, this study aimed to evaluate the incidence and degree of DJUS migration. The study was divided into 2 groups: the radical cystectomy with orthotopic neobladder group and the urinary bladder group. Additionally, it also focused on investigating the risk factors associated with stent migration in the orthotopic neobladder group.

## 2. Materials and methods

### 2.1. Patient characteristics

This retrospective, single-center study was approved by the institutional review board, which waived the need for obtaining informed consent from the patients (approval no. EUMC 2023-07-043). Medical records of patients who underwent antegrade DJUS placement at our institution were retrospectively reviewed. Between July 1, 2021 and March 31, 2023, DJUS were placed via percutaneous nephrostomy (PCN) in 63 consecutive patients. Among them, 37 patients underwent radical cystectomy with an orthotopic neobladder, and 26 patients had a urinary bladder without cystectomy. The inclusion criteria included patients aged 20 to 90 years with urinary obstruction caused by benign UIAS in the orthotopic neobladder group and urinary obstruction due to malignant or benign diseases in the urinary bladder group, and overt hydronephrosis documented on computed tomography (CT). The exclusion criteria were an expected patient life expectancy of less than 3 months, prior kidney transplantation, and poor general health status (Eastern Cooperative Oncology Group performance status grade 4). The patient characteristics are presented in Table 1.

**Table 1:** Baseline demographics and clinical data of the study patients.

Characteristics	Orthotopic neobladder (n = 45)	Urinary bladder (n = 35)	*P*
Sex (%)			.001
Male	39 (86.7)	18 (51.4)	
Female	6 (13.3)	17 (48.6)	
Mean age, yr (range)	69 ± 8.4	65 ± 16.2	.330
Underlying malignancy (%)			<.001
Bladder cancer	45 (100)	16 (45.7)	
Ureter cancer	0	4 (11.4)	
Cervical cancer	0	2 (5.7)	
Prostate cancer	0	1 (2.9)	
Benign ureter stricture	0	8 (22.9)	
Others*	0	4 (11.4)	
Side of obstruction			.383
Right	15 (33.3)	15 (42.9)	
Left	30 (66.7)	20 (57.1)	
Size of DJUS			.538
6-F	30	21	
8-F	15	14	
Type of DJUS			.461
Flexima	12	12	
Inlay optima	33	23	
Balloon dilatation			.383
Yes	30	20	
No	15	15	

* Includes radiation cystitis (n = 2), neurogenic bladder (n = 1), retroperitoneal fibrosis (n = 1).

DJUS = double-J ureteral stent.

### 2.2. Study endpoints and definitions

**Figure 1.**
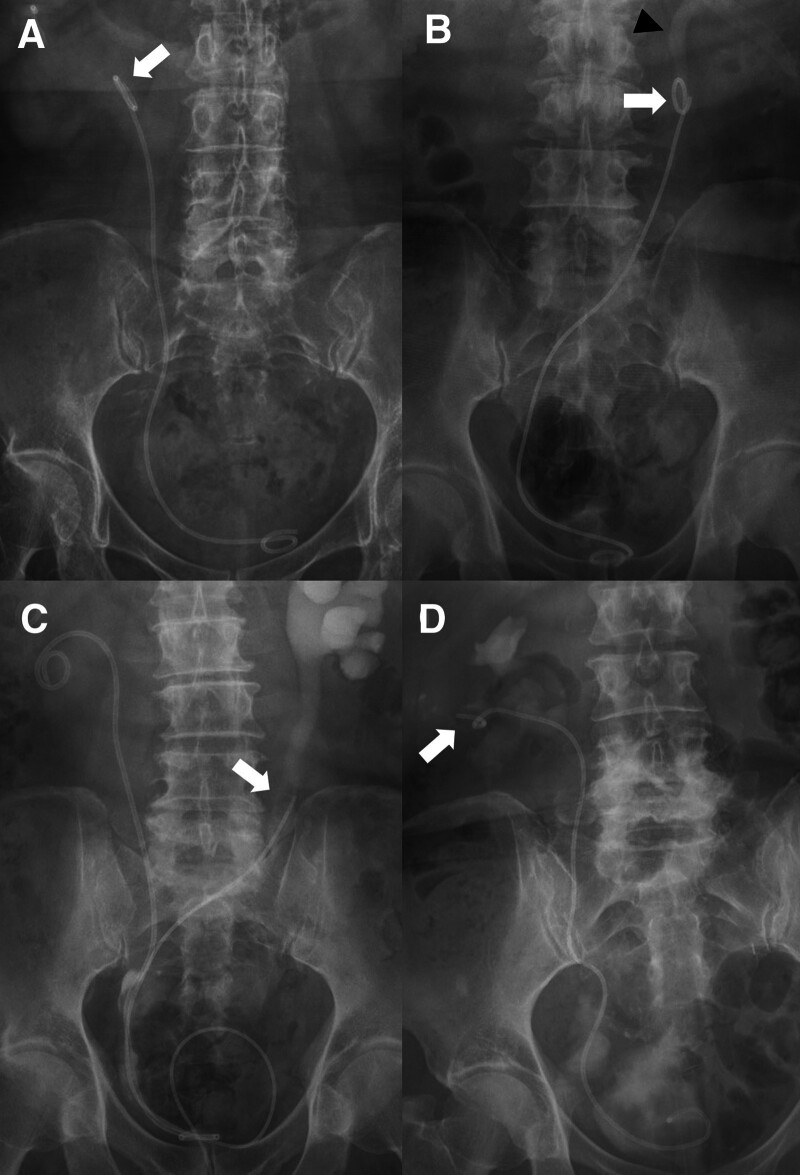
Double-J ureteral stent (DJUS) migration type observed abdomen X-ray. (A) Type I: the proximal tip (arrow) of DJUS located at the ureteropelvic junction. (B) Type II: the proximal tip (arrow) of DJUS located at the proximal ureter (arrowhead). (C) Type III: the proximal tip (arrow) of DJUS located at the mid to distal ureter. (D) Type IV: proximal migration of the proximal tip (arrow) of DJUS.

The patency of the DJUS was confirmed using renal biochemistry or imaging studies, such as ultrasonography or CT scans, at 1 and 3 months after DJUS insertion. Distal migration of the DJUS was defined as a condition in which the proximal tip of the stent no longer resides within the renal pelvis, but rather lies below the ureteropelvic junction (UPJ) or at a lower anatomical level. Proximal migration of the DJUS was defined as a condition in which the proximal tip of the stent was positioned at renal calyx or above and the distal tip of the stent ascended toward the ureter. Stent migration was classified according to the position of the proximal or distal tip of the stent. Type I: the proximal tip is located at the UPJ; Type II: the proximal tip is located at the proximal ureter; Type III: the proximal tip is located at the mid to distal ureter; and Type IV: proximal migration of the stent (Fig. 1). Stent dysfunction was defined as the suspicion of urinary tract infection or hydronephrosis based on either clinical suspicion or imaging studies such as CT or ultrasonography. The following parameters were compared between the 2 patient groups: technical success, stent dysfunction, and complications. Technical success was defined as a successful stent placement at the desired location. Complications were classified as minor or major according to the Society of Interventional Radiology guidelines.^[11]^

### 2.3. Procedure

**Figure 2.**
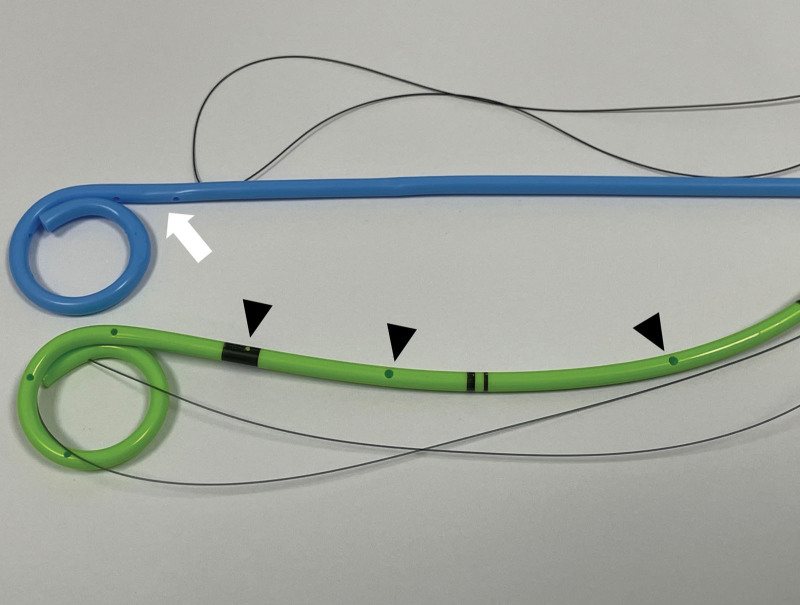
Two type of double-J ureteral stents. 8-F Flexima ureteral stent (Boston Scientific, Natick) with side holes (arrow) in the proximal 2-cm of the straight portion without sideholes in the straight portion (upper). 8-F InLay Optima ureteral stent (BD, Tempe) with multiple side holes (arrowhead) in the straight portion and tip of double-J ureteral stent (lower).

After PCN, an antegrade nephrogram was performed to evaluate the level and length of the obstruction. A 8-F introducer sheath (Pinnacle TIF Tip; Terumo, Tokyo, Japan) was inserted over a 0.035-inch hydrophilic stiff guidewire (Radifocus; Terumo) after removing the PCN tube. A 5-F catheter (Kumpe; Cook, Bloomington, IN, USA) was inserted over the guidewire to traverse the obstructed ureter into the urinary bladder or orthotopic neobladder. Balloon dilatation was subsequently performed using a 3 to 6 mm balloon catheter (Mustang, Boston Scientific, Natick, MA) in case of significant stenosis or occlusion that made it difficult for the guidewire or catheter to pass through. Finally, a 6- or 8-F, DJUS (Flexima; Boston Scientific, Natick, MA, or Inlay Optima; BD, Tempe, Arizona) was inserted with the proximal end in the renal pelvis and distal end within the urinary bladder or orthotopic neobladder. The Flexima ureteral stent had 3 side holes in the proximal 2-cm of the straight portion, whereas the InLay Optima ureteral stent had multiple side holes in the straight portion (Fig. 2).

After stent placement, another PCN tube was inserted to determine the patency of the stent, which was subsequently clamped when the drained urine was clear or showed mild hematuria. Finally, the PCN tube was removed 2 days after the procedure if the nephrogram confirmed good patency and satisfactory positions of the stents, or if patients did not demonstrate any noteworthy symptoms or signs, such as flank pain, fever, or urine leakage via the PCN tract.

### 2.4. Follow-up

All the patients were clinically evaluated 3 months post-procedure using renal biochemistry tests (e.g., blood urea nitrogen or serum creatinine), plain abdominal radiography, and abdominal CT scans. Patients presenting with unexpected symptoms, such as urinary frequency, flank pain, dysuria, hematuria, or fever, were urgently evaluated. DJUS removal was performed if no significant clinical, laboratory, and imaging findings were observed after 3 months. However, in cases where a residual stricture was suspected, retrograde replacement of the DJUS was performed under cystoscopy.

### 2.5. Statistical analysis

All data were accessed for research purposes at June 30, 2023. Continuous variables are presented as mean ± standard deviation and were compared using Student *t* test. Categorical variables were compared using the chi-squared test or Fisher exact test. Univariate and multivariate logistic regression analyses of the risk factors for DJUS migration in patients with orthotopic neobladders were performed. Statistical significance was set at *P* < .05. Statistical analysis was performed using the Statistical Package for the Social Sciences (SPSS 21.0 version) software (IBM Corp, Armonk, NY).

## 3. Results

**Figure 3.**
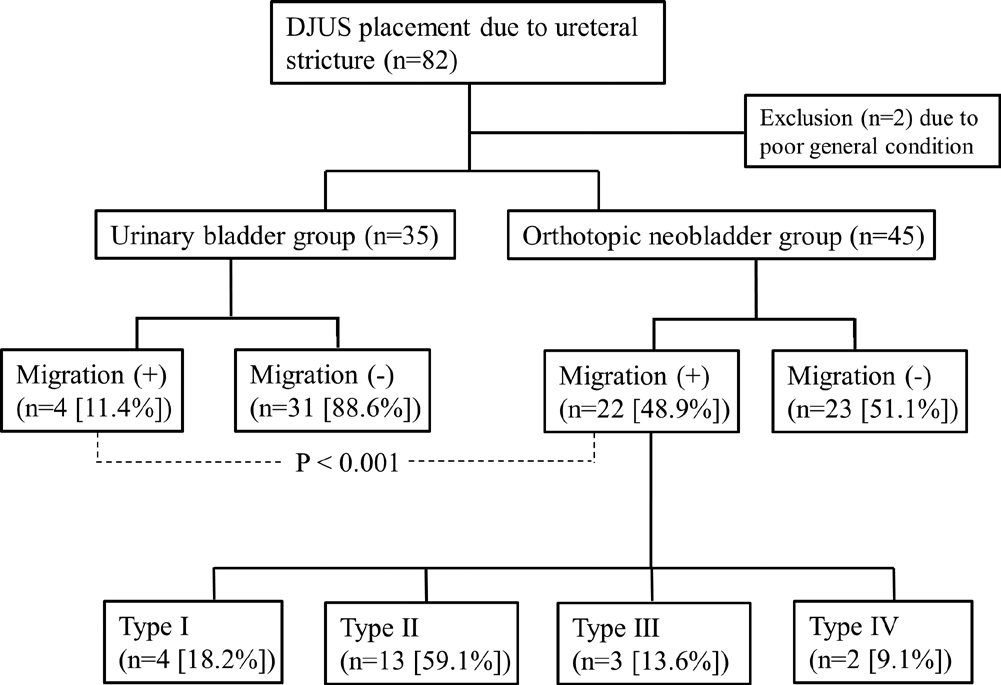
Flowchart of double-J ureteral stent insertion cases in urinary bladder group and orthotopic neobladder group.

The technical success rate was 100% in each group, including 45 DJUS in 35 patients in the orthotopic neobladder group and 35 DJUS in 26 patients in the urinary bladder group. Two patients in the orthotopic neobladder group were excluded as their general condition deteriorated to the point of expiration within a few days after the insertion of DJUS. Stent migration was observed in 22 of 45 cases (48.9%) and 4 of 35 cases (11.4%) in the orthotopic neobladder and urinary bladder groups, respectively. The DJUS migration rates between the 2 groups were statistically significant (*P* < .001) (Fig. 3). When comparing the position of the proximal tip of the DJUS in the immediate post-stent insertion and follow-up plain X-ray, the orthotopic neobladder group demonstrated a change of 2.5 ± 2.8 cm, while the urinary bladder group demonstrated a change of 0.4 ± 1.5 cm (*P* < .001). However, the duration of stent maintenance between the 2 groups was not statistically significant (orthotopic neobladder group: 45.5 days, urinary bladder group: 63.6 days; *P* = .138).

**Figure 4.**
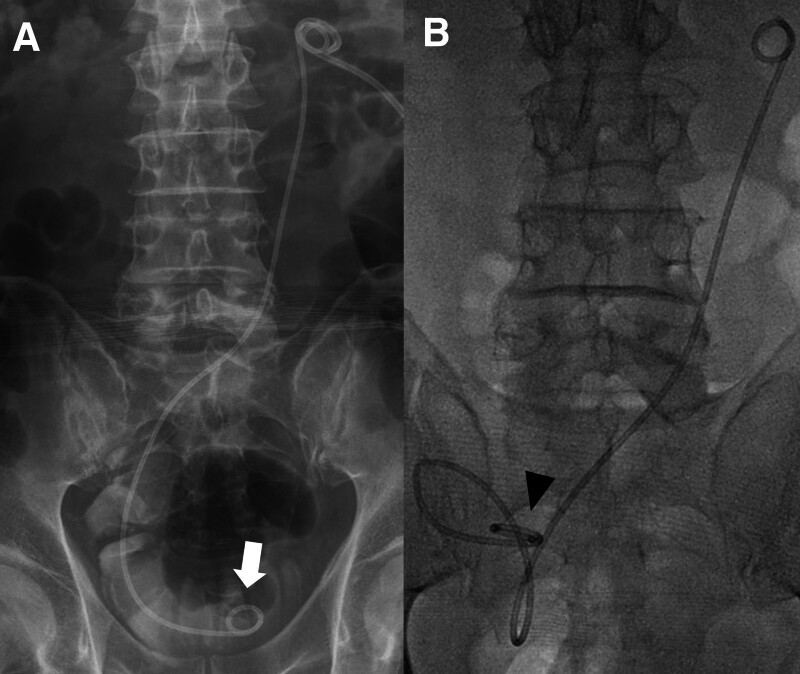
Seventy-seven-yr-old man with bladder cancer and undergone radical cystectomy and orthotopic neobladder. (A) Abdomen X-ray obtained just after placement of InLay Optima and showed proper located distal tip (arrow) of double-J ureteral stent (DJUS). (B) In 2 wk after placement of DJUS, abdomen X-ray showed proximal migration of the distal tip (arrowhead) of DJUS near ureteroileal anastomosis site.

Among the cases with migration in the orthotopic neobladder group, 20 cases (90.9%) had showed distal migration of the proximal tip of the DJUS. Among the 20 cases with distal migration, 4 cases exhibited migration to the UPJ (type I), 13 cases showed migration to the proximal ureter (type II), and 3 cases demonstrated migration to the mid-ureter (type III). Among the 2 cases corresponding to type IV, 1 case exhibited proximal migration of the proximal tip-dislodged renal calyx. Additionally, 1 case revealed proximal migration of the distal tip near the ureteroileal anastomosis site (Fig. 4).

Among the patients in the orthotopic neobladder group who experienced DJUS migration, stent dysfunction occurred in 18 cases (81.8%), which was statistically significant (*P* = .003). Among them, 3 (13.6%) presented with flank pain and accompanying symptoms of infection, necessitating the insertion of a PCN tube. In the remaining 15 cases, follow-up CT scans indicated the presence of persistent hydronephrosis, with 8 cases exhibiting concurrent imaging findings suggestive of urinary tract infections, such as ureteritis. In 4 cases, no specific abnormalities were observed in the clinical, laboratory, or imaging examinations.

In univariate logistic regression analysis, size and type of DJUS were risk factors associated DJUS migration in orthotopic neobladder group (size of DJUS - odds ratio and confidential interval [OR(95%CI)]: 13.000 (2.445–69.131), *P* = .003 in 6-F, type of DJUS—OR(95%CI): 4.615 (1.049–20.306), *P* = .043 in Inlay Optima). However, only size of DJUS was only independent risk factor associated with stent migration in multivariate logistic regression analysis (OR(95%CI): 10.214 (1.749–59.651), *P* = .010) as presented in Table 2. Except for migration, only minor complications, such as self-limiting hematuria (n = 11) and ureter injury during PCN tube insertion (n = 3), were observed; no major complications were observed in any of the patients.

**Table 2:** Multivariable logistic regression analysis of risk factor for double-J ureteral stent (DJUS) migration in orthotopic neobladder patients.

Characteristics	Univariate		Multivariate	
	OR (95% CI)	*P*	OR (95% CI)	*P*
Sex				
Male	0.429 (0.070–2.620)	.359		
Female				
Age				
<60	1			
60–69	0.200 (0.019–2.162)	.185		
70–79	0.250 (0.023–2.696)	.253		
<80	0.125 (0.005–3.225)	.210		
Side of obstruction				
Right	0.583 (0.166–2.052)	.401		
Left				
Size of DJUS				
6-F	13.000 (2.445–69.131)	.003	10.214 (1.749–59.651)	.010
8-F				
Type of DJUS				
Flexima	4.615 (1.049–20.306)	.043	1.909 (0.380–9.590)	.432
Inlay optima				
Balloon dilatation				
Yes	0.510 (0.145–1.798)	.295		
No				

## 4. Discussion

In patients who have undergone radical cystectomy and orthotopic neobladder surgery, the occurrence of UIAS is primarily benign, with <1% attributable to tumor recurrence.^[12]^ The exact underlying causes remain unclear; however, it is postulated that they may be due to periureteral fibrosis and scarring resulting from ischemia or urine leakage at the anastomotic site.^[13,14]^ Although surgical repair demonstrates a high success rate and prolonged patency, it often poses challenges owing to dense adhesions resulting from previous major surgeries or fibrosis associated with radiation therapy.^[15]^ Advancements in interventional radiologic techniques have resulted in the introduction of alternative treatment, such as balloon dilation and ureteral stent placement. Although none of these techniques have achieved long-term patency rates comparable to those of surgical repair, they offer advantages, such as reduced morbidity, shorter hospital stays, and decreased costs.^[16]^

In this retrospective study, we demonstrated a significant increase in DJUS migration in patients who underwent orthotopic neobladder surgery following cystectomy compared to those with a natural urinary bladder. We employed 2 commonly used ureteric stents: Flexima and Inlay Optima. These stents are made of hydrophilic materials and coated with a gel over a copolymer material, facilitating smooth passage within the ureter and reducing encrustation and infection. Although the proximal and distal tips of the stent are designed in a pigtail shape to prevent migration, the disadvantage of using hydrophilic-coated stents is that they may increase the risk of migration.^[9,17,18]^

In the present study, the frequency of distal migration was significantly higher in patients who underwent radical cystectomy and orthotopic neobladder surgery. Several possible explanations exist for these results. First, a normal urinary bladder is structurally designed to support the distal portion of the stent within a limited space, whereas no supporting structure exists for the orthotopic neobladder. This absence of a supporting structure, combined with ureteral and bowel peristalsis, could enhance downward migration. Second, the etiology may vary owing to the underlying disease in the patients. In this study, 65.7% of the urinary bladder group had DJUS inserted owing to malignant obstruction, whereas in patients who underwent radical cystectomy with orthotopic neobladder, the main reason was benign stricture at the anastomosis site. Malignant obstruction is typically caused by cancerous conditions originating from organs external to the urinary system, such as colorectal or gynecological tumors, or from organs within the urinary system, such as bladder and prostate cancers.^[19,20]^ Although no studies have compared stent migration between benign strictures and malignant obstructions, it is presumed that malignant tumors exert stronger forces owing to severe external compression or intrinsic tumor encasement of the stent, which may help prevent migration more effectively than benign strictures such as UIAS.

Univariate logistic regression analysis indicated that both the type (odds ratio [OR]: 4.615, *P* = .043) and size (OR: 13.000, *P* = .003) of the DJUS were significant factors promoting migration in the orthotopic neobladder group. Perhaps 2 different types of stents were used, and the Inlay Optima, which has multiple side holes in the straight portion, might produce more friction in the stricture part, potentially causing less migration. However these disparities did not yield significant results in the multivariate logistic regression analysis (OR: 1.909, *P* = .432). Given the small sample size, the actual impact of these factors remains uncertain. Therefore, future studies involving larger patient cohorts are warranted.

In the multivariate logistic regression analysis, only DJUS size was identified as a significant risk factor (OR: 10.214, *P* = .010). The 6-F stent has only 75% of the external lumen compared with the 8-F stent, resulting in weaker support against extrinsic compression at the UIAS site. Docimo and DeWolf noted a high failure rate in patients with extrinsic ureteral obstruction, often caused by tumor compression, when small stents (6-F) were used.^[9,21]^ However, in this study, when only patients with 6-F stents were compared, those in the migration group showed statistically significant stent dysfunction (OR: 5.667, *P* = .006). Therefore, in the case of orthotopic neobladders, smaller stent sizes may also contribute to early dysfunction, and an increased propensity for migration may lead to stent dysfunction.

This study had several limitations. First, it was retrospective in nature, which prevented control over certain conditions, such as the day of PCN placement, the time between DJUS and PCN insertion, and the day of follow-up. Second, the male-to-female ratio in the cystectomy with orthotopic neobladder group was approximately 6.5 to 1. However, the incidence of bladder cancer is 3 to 4 times higher in men than in women.^[22]^ Future studies should take this into consideration and ensure necessary adjustments of the variables. Finally, this was the first study to investigate DJUS migration in orthotopic neobladders. Therefore, further studies are warranted to explore techniques and devices for preventing stent migration.

In conclusion, when UIAS occurs in patients undergoing radical cystectomy and orthotopic neobladder, DJUS migration can occur easily. Smaller stent sizes are associated with a higher incidence of migration; thus, the size of the stent should be taken into consideration during the procedure. Further research is warranted to prevent migration in the future.

## Author contributions

**Conceptualization:** Chang Hoon Oh, Soo Buem Cho, Hyeyoung Kwon.

**Data curation:** Chang Hoon Oh.

**Investigation:** Hyeyoung Kwon.

**Methodology:** Chang Hoon Oh.

**Supervision:** Yeok Gu Hwang.

**Writing – original draft:** Chang Hoon Oh, Hyo Jeong Lee.

**Writing – review & editing:** Chang Hoon Oh, Soo Buem Cho.
